# Effector T Helper Cell Subsets in Inflammatory Bowel Diseases

**DOI:** 10.3389/fimmu.2018.01212

**Published:** 2018-06-01

**Authors:** Tanbeena Imam, Sungtae Park, Mark H. Kaplan, Matthew R. Olson

**Affiliations:** ^1^Department of Pediatrics and Herman B Wells Center for Pediatric Research, Indiana University School of Medicine, Indianapolis, IN, United States; ^2^Department of Biological Sciences, Purdue University, West Lafayette, IN, United States

**Keywords:** inflammatory bowel disease, Crohn’s disease, ulcerative colitis, T helper cells, inflammatory cytokines, transcription factors

## Abstract

The gastrointestinal tract is a site of high immune challenge, as it must maintain a delicate balance between tolerating luminal contents and generating an immune response toward pathogens. CD4^+^ T cells are key in mediating the host protective and homeostatic responses. Yet, CD4^+^ T cells are also known to be the main drivers of inflammatory bowel disease (IBD) when this balance is perturbed. Many subsets of CD4^+^ T cells have been identified as players in perpetuating chronic intestinal inflammation. Over the last few decades, understanding of how each subset of Th cells plays a role has dramatically increased. Simultaneously, this has allowed development of therapeutic innovation targeting specific molecules rather than broad immunosuppressive agents. Here, we review the emerging evidence of how each subset functions in promoting and sustaining the chronic inflammation that characterizes IBD.

## CD4^+^ T Cells in Inflammatory Bowel Disease (IBD)

Inflammatory bowel disease is a complex set of diseases that includes Crohn’s disease (CD) and ulcerative colitis (UC), each with multiple bacterial, immune, and non-immune cell types contributing to inflammation. However, there are a number of lines of evidence that suggest that CD4^+^ T helper cells are major initiators in the disease process. CD4^+^ T cells are enriched in lesional tissue from patients with CD and UC and blockade or depletion of CD4^+^ T is effective in treating patients with IBD. In these studies, CD4^+^ T cell-depleting and blocking antibodies caused remission from disease in a number of CD and UC patients examined, suggesting a prominent role of CD4^+^ T cells in propagating disease (1, 2). Interestingly, IBD patients with concurrent HIV infection also exhibit a greater incidence of remission as compared to non-HIV controls, correlating with decreased total blood CD4^+^ T cell counts (3, 4). Finally, a number of biologics that target CD4^+^ T cell differentiation into inflammatory subsets or their byproducts (i.e., cytokines) have shown efficacy in treating patients with IBD (5–7). Given the importance of CD4^+^ Th cells in the disease process, this review will focus on how Th cells differentiate in the inflamed intestinal tract during IBD and how the Th lineage-specific cytokines and transcription factors (TFs) contribute to disease.

## Th1 Cells

**Figure 1:**
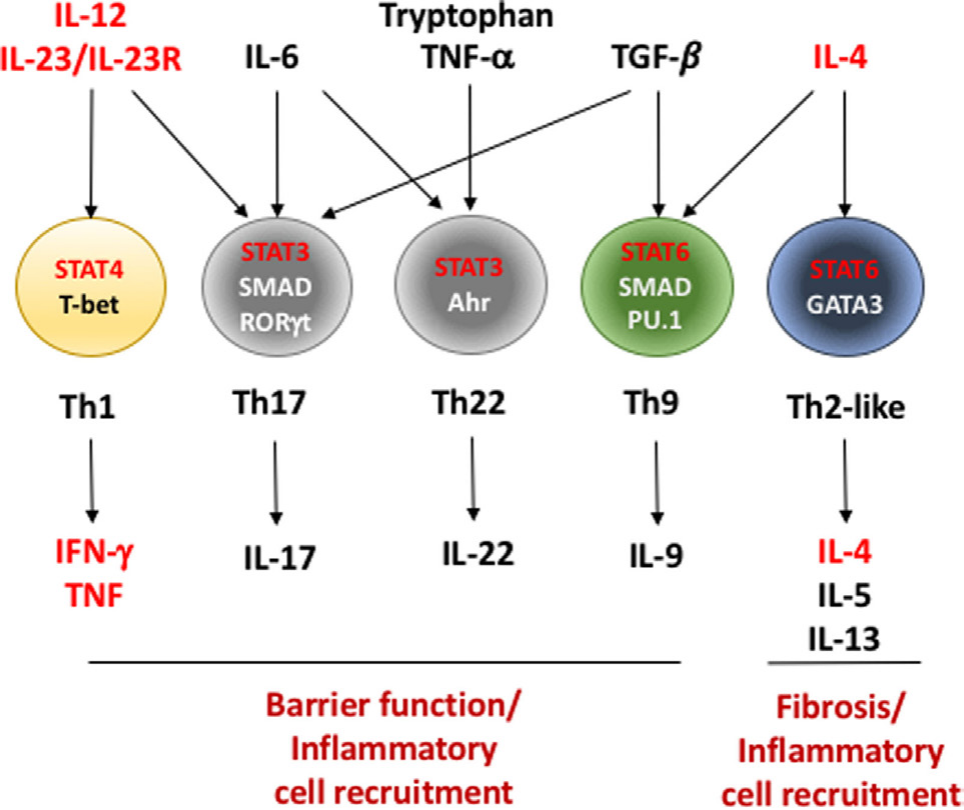
Critical factors in the differentiation of effector Th cells during inflammatory bowel disease (IBD). T helper cells recognize antigen presented in the context of major histocompatibility complex II on antigen-presenting cells in a T cell receptor-dependent fashion (not shown). In conjunction with assorted co-stimulatory signals (i.e., CD80/86–CD28 interaction and others), these signals initiate a program of cell division and differentiation. This differentiation program can be profoundly influenced based on the cytokines present in the environment in which they are initiated. Interleukin (IL)-12 and IL-23, cytokines induced during early stages of IBD, play important roles in differentiation of interferon (IFN)-γ/tumor necrosis factor (TNF)-producing Th1 cells as well as IL-17-producing Th17 cells. However, Th17 cells require additional signals including IL-6 and TGF-β for full induction of their differentiation. IL-6, in combination with TNF-α and tryptophan metabolites, initiates differentiation of protective IL-22-producing Th22 cells. Th1 differentiation is initiated and stabilized by transcription factors signal transducer and activator of transcription (STAT)4 and T-bet while Th17 cells require a combination of transcriptional regulators including STAT3, SMAD proteins, and RORγt. IL-4, IL-5, and IL-13-secreting Th2 and IL-9-secreting Th9 cells require IL-4 and STAT6 for their differentiation. Similar to Th17 cells, Th9 cells additionally require TGF-β, SMAD proteins, and a TGF-β/SMAD-induced transcription factor PU.1 for their development. As a whole, the inflammatory mediators produced by Th cells in IBD play a role in the maintenance or breaking down gut epithelial barriers or in recruiting unique cells types to the intestines that further promote inflammation. Factors in red indicate genes involved in Th cell differentiation or function that contain single nucleotide polymorphisms that are associated with increased disease susceptibility or severity in humans (see Table 2).

Th1 cells are important for protecting against infectious pathogens. These cells primarily produce interferon (IFN)-γ and tumor necrosis factor (TNF) that, respectively, activate macrophages and direct cytotoxic CD8^+^ T cell responses, that in turn promote elimination of intracellular pathogens such as viruses and bacteria (8). In IBD, however, Th1 cells accumulate in the intestinal tract of individuals with CD and are directly associated with disease. Interleukin (IL)-12, which is secreted by antigen-presenting cells, acts *via* signal transducer and activator of transcription (STAT)4 to promote the differentiation of naïve T cells into Th1 cells (9–11). STAT4 also signals activation of the TF T-bet, a lineage-defining factor for Th1 differentiation, which upregulates the IL-12 receptor, IFN-γ expression, and causes further expansion of Th1 cells (Figure 1) (12).

### Th1-Associated Cytokines

#### Interferon-γ

Interferon-γ is the defining cytokine produced by Th1 cells and is used almost exclusively to identify Th1 cells in settings of disease. During intestinal inflammation, IFN-γ in combination with another Th1-associated cytokine, TNF, was proposed to drive intestinal epithelial cell beta catenin signaling and limit their differentiation and proliferation (13). Despite this proposed model, the role of IFN-γ in murine IBD is controversial. Powrie et al. (14) and Ito et al. (15) both demonstrate that IFN-γ is required for disease development in a CD45RB^hi^ RAG adoptive transfer model and in a DSS model of IBD (see Table 1), respectively. Nava et al. (13) also observed moderately reduced disease in IFN-γ-deficient mice in a DSS model of disease. Loss of IFN-γ in these reports correlated with overall reduced inflammation and tissue damage as well as reduced type-1-associated chemokine expression that would recruit other inflammatory cells to the intestinal tract. In contrast to these studies, Simpson et al. (16), using a adoptive transfer model of colitis modified from Powrie et al. (14), demonstrated that IFN-γ was not required for disease in this setting. Furthermore, Muzaki et al. (17) showed that IFN-γ-deficient mice were in fact more susceptible to DSS-induced colitis, suggesting a protective role for IFN-γ. In a TNBS model of colitis (Table 1), IFN-γ was neither protective nor required for disease (18, 19).

**Table 1:** Models of Th cell-driven inflammatory bowel disease.

Mouse model	Type of model	Th-type/s involved	Key cytokines/TFs	Reference
DSS colitis	Chemical	Th1 (Acute)	IFN-γ, TNF, T-bet	(13, 15, 20–22)
		Th2 (Chronic)	IL-4, IL-13, STAT6, GATA3	
		Th9	IL-9, PU.1	

TNBS colitis	Chemical	Th1 (Acute)	TNF, T-bet	(23–30)
		Th2 (Chronic)	IL-13, GATA3	
		Th9 (Acute)	IL-9, PU.1	
		Th17 (Acute)	IL-17R, SMAD7	

Oxazolone	Chemical	Th2, NK-T	IL-4, IL-13	(29, 31–35)
		Th9	IL-9, GATA3	
		Th17	TGF-β, SMAD7	

Anti-CD40	Chemical (activating antibody)	Th1	IL-12	(36)
		Th17	IL-17, IL-23	

Citrobacter rodentium	Infectious	Th1	IFN- γ	(37, 38)
		Th17	IL-17A, IL-17F	

RAG or SCID T cell transfer model	Transfer	Th1	IFN-γ, STAT4, T-bet	(14–16, 23, 39–41)
		Th17	IL-23, STAT3, SMAD2	

*Abcb1an*^−/−^	Transgenic	Th17	IL-23	(42)

*Tcrα*^−/−^	Transgenic	Th2-like	IL-4	(43)

*Il10/Il10r*^−/−^	Transgenic	Th17	IL-17, IL-23	(44)

*Th-association was determined by induced expression in a particular model or by mechanistic studies, where cytokines/TFs were manipulated by gene deletion or blocking antibody*.

*Acute, acute exposure to chemical; Chronic, repeating exposure to chemical; STAT, signal transducer and activator of transcription; IL, interleukin; TNF, tumor necrosis factor; IFN, interferon; TFs, transcription factors*.

The seemingly contradictory nature of the above studies suggests that other factors may be involved or compensate for the loss of IFN-γ in some models. Alternatively, the dependence of disease on IFN-γ may be a factor of the exact model system used or the differences in gut microbiomes within populations of mice across institutes or may be attributable to different mouse strains used. However, in human GWAS studies, there is a clear enrichment of SNPs in a CD- and UC-associated risk area comprised of several regions up and downstream of the human *IFNG* gene (45, 46). Furthermore, a particular IBD-associated SNP within the *IFNG* gene (rs1861494) is functionally linked with elevated IFN-γ expression in IBD patients (Figure 1; Table 2) (47). These data suggest at least a possible role of IFN-γ in promoting IBD in humans.

**Table 2:** Single nucleotide polymorphisms (SNPs) associated with Th-associated cytokines or transcription factors.

Gene	SNP	Th subset association	Population studied	Disease type	Reference
*IL12B*	rs3212227	Th1	– Caucasian – Spanish	Crohn’s disease (CD), ulcerative colitis (UC)	(48–50)
	rs6887695	Th1	– Spanish	CD, UC	(49)
	rs2288831	Th1	– Korean	CD	(51)
	rs10045431	Th1	– British (Caucasian)	CD, UC	(52, 53)
	rs6871626	Th1	– British (Caucasian)	CD, UC	(54)
	rs6556412	Th1	– British (Caucasian)	CD, UC	(54)

*IFNG*	rs1861494	Th1	– Caucasian	CD, UC	(47)
	rs7134599	Th1	– British (Caucasian)	UC	(54)

*TNF*	rs1800629	Th1	– Spanish – Portuguese	Inflammatory bowel disease	(55, 56)
	rs1799964	Th1	– Iranian – French Canadian	CD	(57, 58)

*STAT4*	rs7574865	Th1	– Spanish	UC	(59)
	rs925847	Th1	– Korean	UC	(60)

*IL23R*	rs11805303	Th17	– British (Caucasian)	CD, UC	(48)
	rs11209026	Th17	– Dutch(Caucasian), – Hungarian(Caucasian), – New Zealanders(Caucasian), – British (Caucasian) – Spanish	CD, UC	(49, 61–64)
	rs7517847	Th17	– British (Caucasian) – Spanish	CD	(49, 65)
	rs1004819	Th17	– German/Caucasian	CD	(50)
	rs11465804	Th17	– Mixed	CD	(52)

*STAT3*	rs12948909	Th17	– British (Caucasian) – German (Caucasian)	UC	(53, 66, 67)
	rs744166	Th17	– Caucasian	CD, UC	(67)

*JAK2*	rs10758669	Th17	– British (Caucasian) – Mixed	CD, UC	(52–54)

*STAT6*	rs324015	Th2/Th9	– British (Caucasian)	CD	(68)

*IL4*	rs2243250	Th2/Th9	– Iranian	CD, UC	(69)
	rs2243248	Th2/Th9	– Iranian	CD, UC	(69)

#### Tumor Necrosis Factor

A wide array or both immune (i.e., Th1 cells, Figure 1) and non-immune cells produce TNF during inflammation that induces signaling through two distinct receptors, TNFR1 and TNFR2. Similar to IFN-γ, TNF has also been demonstrated to be involved in intestinal barrier dysregulation during IBD (13) and has a varied role in mouse models of intestinal inflammation. Blockade of TNF in mice undergoing TNBS-induced disease, exhibited reduced weight loss and inflammation (24). Similarly, Yang et al. (25) demonstrated that TNFR1-, TNFR2-, and TNFR1/TNFR2 double-deficient mice exhibited similarly reduced TNBS-induced IBD as compared to control mice, suggesting a non-redundant role each receptor in this model. However, these findings were challenged by other studies in the TNBS model system where Ebach et al. (70) demonstrated that TNFR1-deficient mice had exacerbated disease and TNFR2-deficient mice had attenuated disease compared to controls, suggesting a protective and exacerbating role for each receptor, respectively.

Strikingly similar to the role of IFN-γ in murine IBD, the effect of TNF also varies greatly between model systems. Despite the protective role of TNF blockade in the TNBS model, TNF blockade in oxazolone-treated mice had little effect (24). In addition, Stillie and Stadnyk (71) showed that both TNFR1 and TNFR2-deficient mice developed significant disease after DSS treatment. Even more surprisingly, Wang et al. (72) reported exacerbated disease in DSS-treated TNFR1- and TNFR2-deficient mice as compared to controls, suggesting a protective role of TNF signaling during murine IBD. In these last experiments, lack of TNF signaling resulted in production of another auto-immune-associated cytokine, GM-CSF that played a critical role in the exacerbated disease phenotype, suggesting a role for compensating cytokines in some of these model systems. As a whole, these data indicate a need for standardization of model systems and sharing of reagents between laboratories to uncover potential discrepancies in these studies.

In humans, there are several SNPs in the TNF gene that are associated with IBD in a number of populations (Figure 1; Table 2). In addition, anti-TNF therapy is currently used in treatment of both CD and UC and is effective in a number of patients. Interestingly, while anti-TNF therapy was sufficient for complete remission of IBD-related symptoms (5, 6, 73), only ~24–50% of patients exhibited mucosal wound healing depending on the type of anti-TNF antibody used (74, 75). These data indicate a disconnect between tissue damage and what generally makes IBD patients feel “sick” (i.e., possibly more systemic effects). While both factors are relevant end points, mucosal wound healing is potentially more meaningful as these patients may be less likely to relapse after ceasing anti-TNF therapy.

### Th1-Associated TFs

#### T-bet

T-bet is required for the differentiation of Th1 cells both *in vitro* and *in vivo* in the context of IBD. *Tbx21*-deficient CD4 T cells, isolated from BALB/c mice, were unable to induce colitis in a SCID adoptive transfer model of IBD. Furthermore, overexpression of T-bet in Th cells exacerbated experimental colitis in this same model (23). By contrast, a more recent study demonstrates that *Tbx21*-deficient CD4 T cells, this time from C57BL/6 mice, produced similar pathology as WT CD4 T cells (76). However, while *Tbx21*-deficient cells exhibited a dramatic decrease in IFN-γ-production, there was a corresponding increase in the proportion of cells that produced IL-17 and other Th17-associated cytokines in the inflamed intestines. Further mechanistic work demonstrated that *Tbx21*-deficient cells had heightened sensitivity to IL-23 signaling and increased RORγt expression, a TF associated with Th17 differentiation. As a whole, these data demonstrate that T-bet plays a pivotal role in Th1 differentiation in murine models of IBD, however, in some cases, the loss of Th1 cells is compensated for by an increase in pathogenic IL-17-producing cells resulting in a similar disease phenotype.

T-bet can also play a protective role in murine IBD. *Tbx21*-deficient mice develop more severe colitis than control animals after treatment with DSS. This elevated disease is marked by enhanced immune cell infiltration into the intestines, ulceration, and loss of crypts (77). Interestingly, this disease became a spontaneous disease when crossed to Rag2-deficient mice that lack T and B cells, suggesting that CD4^+^ T cells do not drive this disease. Instead, Garrett et al. (77) and Powell et al. (78) demonstrated that colitis in these mice was induced by exaggerated TNF production in dendritic cells (DCs) and elevated numbers of IL-17-producing innate lymphoid cells (ILCs) in the intestinal tract that causes a microbial breach in the intestinal lining.

Human GWAS studies have identified several SNPs that were associated with development of UC and CD (46, 79). Interestingly, a number of these SNPs sites were enriched for T-bet binding. Although T-bet bound a number of these intergenic regions in healthy individuals, several of these SNPs lead to reduced T-bet binding at these sites and altered gene expression of a number of Th1 related factors (*IL18RAP and TRIB1*) (80). Although both IL-18 and IL18RAP have been previously shown to be also associated with IBD, the role of TRIB1 in this setting is unclear. While these data need to be further verified using *in vivo* models of IBD, it might suggest that T-bet serves more of an inflammatory role than a protective role in human disease.

#### Signal Transducer and Activator of Transcription 4

Binding of IL-12, produced by DCs and other innate immune cells, to its receptor triggers the phosphorylation of STAT4 which then translocates to the nucleus and initiates the transcriptional network associated with Th1 differentiation (81, 82). STAT4 binds a number of distinct regions within the genome including a number of key Th1-associated genes [i.e., *Ifng* and *Tbx21* (82, 83)]. Given its initiator role, STAT4-deficiency leads to a major impairment of Th1 differentiation *in vitro* as well as in mouse models of IBD (9, 10, 16). In support of these initial findings, mice engineered to overexpress STAT4 (STAT4-transgenic mice) developed an IBD-like disease after administration of dinitrophenyl-conjugated keyhole limpet hemocyanin that correlated with increased numbers of IFN-γ and TNF-producing CD4^+^ T cells in the intestines (84). Interestingly, IFN-γ, a key Th1-associated gene downstream of STAT4 signaling, was not required for disease in an adoptive transfer model of IBD (16). These data suggest that although STAT4 is critical for Th1 differentiation and disease development in murine IBD, it may promote disease IFN-γ-independent manner.

Interleukin-12 and STAT4 also appear to be linked to IBD in humans. There are a number of IBD-associated SNPs in the *IL12B* gene in Caucasian populations that correlate with enhanced disease (Figure 1; Table 2). STAT4 SNPs are associated with UC, but not CD, in some populations (51, 59, 85), suggesting a restricted role for these polymorphisms in human disease. More strikingly, STAT4 isoform ratios appear to be a better predictor of disease in IBD patients. STAT4 is produced as two distinct isoforms (α and β), where the STAT4β variant lacks 44 amino acids at the C-terminal end of the protein spanning the transactivation domain. Patients with UC or CD have elevated STAT4β:STAT4α ratios as compared to non-IBD controls and this ratio is normalized after steroid treatment (86). In both murine models of colitis and experimental autoimmune encephalomyelitis, CD4 T cells only expressing the STAT4β isoform drive exacerbated disease as compared to cells expressing the STAT4α isoform (87, 88). In the murine IBD model, STAT4α- and STAT4β-expressing cells had a similar capacity to produce IFN-γ and IL-17, but STAT4β preferentially drove expression of TNF and GM-CSF that have been also described to play a role in IBD severity (25, 88). Again, these data indicate that while STAT4 in CD4 T cells is critical for development of IBD, it might do so independently of IFN-γ.

## Th17 Cells

The traditional Th1/Th2 paradigm of CD and UC was challenged with the discovery of a unique subset of IL-17-producing, termed Th17 cells (89). Th17 cells play an important role in maintaining commensal populations at important barrier sites (i.e., skin and gut), but when triggered in settings of autoimmunity can often exacerbate disease (89). The Th17 differentiation program is driven primarily by IL-6 and TGF-β (90) and further stabilized by signals from accessory cytokines including IL-23 and IL-1β (Figure 1) (91, 92). Interestingly, Th17 differentiation is inhibited by cytokines produced by other Th lineages including IFN-γ, IL-4, and IL-2 (93, 94). As IL-6 induces STAT3 phosphorylation, STAT3 and other downstream TFs, retinoic-acid-receptor-related orphan receptor (ROR) gamma and ROR alpha, are critical for Th17 differentiation *in vitro* and *in vivo* (95, 96). After differentiation, Th17 cells primarily express IL-17A and IL-17F; however, these cells can also co-produce signature cytokines from other Th cell lineages in particular settings of autoimmune inflammation (i.e., IFN-γ: Th1, IL-22: Th22, IL-9: Th9).

### Th17-Associated Cytokines

#### Interleukin-17

Th17 cells are largely defined by their capacity to produce IL-17 (IL-17A and/or IL-17F) in settings of inflammation. IL-17 signals through a heterodimeric receptor (IL-17RA and IL-17RC) that is expressed on many non-hematopoietic cells including intestinal epithelial cells and on some activated T cells (97). Signaling through these receptors plays an important role in epithelial cell barrier function, and in the production of inflammatory chemokines and cytokines by target cells. In Th cells, IL-17 can also dampen the production of IFN-γ thereby possibly enhancing the stability of the Th17 phenotype by limiting Th1 differentiation (98).

Interleukin-17A protein in the serum and IL-17-producing Th cells in the gut-draining lymph nodes were elevated in patients with CD, but not UC, over non-IBD patients (99). In addition, the numbers of IL-17^+^ cells in the intestines of patients with active CD and UC were also elevated as compared to healthy controls and patients with inactive CD or UC (100). While IL-17A blockade is successful in diminishing disease in patients with plaque psoriasis and ankylosing spondylitis, treatment of CD patients with IL-17A-blocking antibodies surprisingly enhanced disease severity resulting in a premature end to these clinical trials (101, 102). These adverse effect studies were substantiated in mice where IL-17 blockade in a DSS model of colitis exacerbated disease symptoms as well as immune cell infiltration into the mucosa (103). This result was recapitulated in IL-17-deficient mice treated with DSS (104). Furthermore, transfer of IL-17A- or IL-17RA-deficient Th cells into RAG recipient mice lead to an enhanced colitis-like wasting disease (98). Interestingly, IL-17R-deficient and IL-17R-Ig fusion protein-treated mice were protected from disease in TNBS model of UC, suggesting that the protective role of IL-17 may depend on the particular model of colitis used (30).

One explanation for the enhanced disease observed in anti-IL-17A-treated or IL-17A-deficient mice may be compensatory effects of other Th subsets or inflammatory cytokines. Indeed, O’Connor et al. (98) observed increased intestinal *Ifng* mRNA and increased Th1 polarization in the absence of IL-17A. Furthermore, addition of IL-17A to developing Th1 cells in culture suppressed IFN-γ production, suggesting that IL-17A limits Th1 differentiation and Th1-mediated immunopathology. Unfortunately, the authors did not neutralize IFN-γ in these mouse experiments to determine if elevated IFN-γ in mice that had received IL-17A-deficient Th cells was the cause of exacerbated disease. However, in *Abcb1a*-deficient mice that also develop enhanced colitis after *Helicobacter* infection and IL-17RA blockade, co-blockade of IFN-γ did not decrease disease severity (42), suggesting that increased IFN-γ may not be causative for the enhanced disease observed in the absence of IL-17. Leppkes et al. (105) demonstrated a different type of immune compensation in the absence of IL-17A. This study observed striking increases in IL-17F after adoptive transfer of IL-17A-deficient cells into RAG-deficient hosts. Interestingly, antibody blockade of IL-17F in mice that had received IL-17A-deficient Th cells ablated disease. Similarly, Wedebye Schmidt et al. (106) also found that antibody-mediated blockade of either IL-17A or IL-17F alone was insufficient to reduce disease severity, whereas blockade of both IL-17A and IL-17F completely abrogated disease. These studies highlight the compensatory nature of the intestinal immune response during IBD and indicate that multiple Th cell types and cytokines likely work in concert to promote autoimmunity in this setting.

Another theory of why the absence of IL-17A enhances intestinal inflammation lies within its ability to regulate epithelial barrier function and gut homeostasis. Maxwell et al. (42) observed increased gut permeability in the *Abdcb1a*-deficient colitis model when IL-17RA signaling was abrogated *via* blocking antibodies as compared to control mice. This was further substantiated in a DSS model of colitis where antibody-mediated blockade of IL-17 also resulted in enhanced “leakiness” of the intestinal epithelial barrier (107). In both cases, the increase in permeability correlated with changes in epithelial tight junction gene expression and changes in occludin positioning within the damaged epithelial layer (42, 107). Interestingly, Maxwell et al. (42) demonstrated that IL-17 signaling was also critical for production of anti-microbial peptides (AMPs) that may influence bacterial populations within the intestines during colitis. Song et al. (104) also showed that IL-17, in concert with fibroblast growth factor 2, regulated both epithelial barrier function and bacterial homeostasis in the gut. As a whole, these data indicate that although IL-17 has bona fide inflammatory properties, the barrier maintenance and microbial “grooming” function of IL-17 is likely dominant and is crucial for protection against intestinal barrier breach and enhanced inflammation during colitis.

#### Interleukin-23

Interleukin-23 is composed of the IL-12p40 and IL-23p19 subunits and is produced primarily by DCs and monocytes in IBD (108, 109). IL-23 signals through the IL-23 receptor (IL-23R) which is induced by TGF-β, IL-6, and STAT3 signaling on activated T cells (110, 111). IL-23 distinctly enhances Th17 differentiation *in vitro* and lineage commitment *in vivo* (112, 113). Furthermore, IL-23 levels are elevated in intestinal biopsies taken from patients with IBD (109) and SNPs in the IL23R locus have been associated with increased risk for IBD (Table 2) (79, 114).

Interleukin-23 is also elevated in mice with colitis and largely appears to be required for disease progression. Interestingly, IL-23 plays an important role in both innate and adaptive immune-driven disease. Hue et al. (39) demonstrated that treatment with neutralizing antibodies to IL-23p19 blocked the development of innate-driven infectious colitis in a 129SvEvRAG^−/−^ mice. Furthermore, adaptive immune-driven colitis in an adoptive transfer RAG model of disease was abolished when CD45RBhi cells were transferred into mice lacking both IL-12p40 and IL-23p19, but not in mice lacking individual cytokines (39). These findings were confirmed in similar models where either IL-23R-deficient donor cells (115) were transferred or IL-23 signaling was blocked with neutralizing antibodies (116). There was also a reduction in disease in IL-23p19-deficient mice treated with anti-CD40 agonist antibody as compared to anti-CD40-treated WT controls (Table 1) (36). In essentially all situations, reduced disease in these animals correlated with a reduction in IL-17^+^ CD4 T cells, particularly those that co-produce IFN-γ, suggesting that these IL-17^+^/IFN-γ^+^ cells may be the major inducers of disease. Interestingly, both Ahern et al. (115) and Uhlig et al. (36) observed that while IL-23p19-deficient mice lacked intestinal pathology there was very little difference in the weight loss that is often associated with murine models of colitis. By contrast, IL-12p40-deficient mice exhibited reduced weight loss, but minimal reductions in intestinal pathology (36), suggesting that weight loss and intestinal pathology are controlled by divergent mechanisms.

Similar to IL-17, IL-23 is not always pathogenic, and in some cases, can have a protective role. Becker et al. (37) demonstrated in TNBS and DSS models of colitis that IL-23p19-deficient mice were much more susceptible to developing colitis as compared to their WT counterparts. This phenomenon was also observed in a model of infectious colitis, using *C. rodentium*, where antibody-mediated blockade of IL-23 or IL-23-deficiency exacerbated disease and enhanced mortality compared to controls (117). Both Aychek et al. (117) and Becker et al. (37) noted increased IFN-γ in the absence of IL-23 and enhanced disease could be ameliorated by blockade of IL-12 or IFN-γ. This is consistent with several reports noted above where lack of IL-17 may result in enhanced Th1 responses and suggests a delicate balance in mechanisms that control development of intestinal inflammation.

### Th17-Associated TFs

#### Signal Transducer and Activator of Transcription 3

STAT3 is activated *via* phosphorylation by kinases associated with the IL-6 and IL-21 receptor and is critical for *in vitro* and *in vivo* differentiation of Th17 cells (96, 118). In IBD patients, there is an increase in both total STAT3 protein as well as phosphorylated STAT3 in the inflamed colon over non-IBD controls which correlated with disease severity (119, 120). There is also a *STAT3* SNP associated with enhanced IBD susceptibility or disease severity in different populations, further implicating a role for STAT3 in both CD and UC (Figure 1; Table 2) (67, 121, 122). In line with these observations in humans, STAT3 signaling also plays a role in a mouse model of IBD. In a RAG adoptive transfer model of IBD, Durant et al. (40) demonstrated that STAT3-deficient CD45RBhi CD4 T cells were unable to differentiate into IL-17-producing cells and were unable to initiate inflammation in the intestines. Interestingly, lack of STAT3 in CD4 T cells had little effect on their capacity to produce IFN-γ, but significantly enhanced the frequency of these cells that became FOXP3^+^ T regulatory cells. STAT3-deficient CD4 T cells also lacked the ability to proliferate or survive in the lymphopenic environment present in RAG-deficient hosts. The authors further demonstrated *via* CHIP-seq analysis that STAT3 binds and promotes epigenetic remodeling of both Th17- and anti-apoptotic/survival-associated genes suggesting a complex role for T cell-intrinsic STAT3 in the progression of colitis.

#### SMADS

TGF-β binding to its receptor triggers activation and phosphorylation of SMAD proteins that translocate to the nucleus and influence transcription of a variety of genes involved in tumor metastasis and T helper cell differentiation. In T cells, TGF-β induces SMADs 2 and 3 that form a complex with SMAD4 and activate transcription of a number of genes including FOXP3 and RORγt (123). Furthermore, SMAD-induced TGF-β signaling also results in increased expression of SMAD7 that acts as an inhibitor of TGF-β-induced signaling pathway (124).

Although TGF-β is critical in the differentiation of Th17 cells, each SMAD protein has a unique and sometimes antagonizing role in this process. SMAD2 promotes Th17 differentiation *in vitro* by enhancing IL-6R expression or by physically interacting with the Th17-associated TF RORγt (41, 125). Furthermore, SMAD2-deficient CD4 T cells were unable to initiate colitis in a RAG adoptive transfer model and in a *C. rodentium* model of infectious colitis (Table 1) (41). By contrast, SMAD3-deficiency resulted in enhanced Th17 differentiation *in vivo* (125) and SMAD4-deficiency had little effect on Th17 differentiation (96). However, more recent reports indicate that SMAD4 degradation might play an important role in Th17 differentiation. Zhang et al. (126, 127) demonstrated that SMAD4-deficient cells produced IL-17 in the absence of TGF-β in culture, an over-looked point in the previous study, and ectopic SMAD4 expression suppressed Th17 differentiation. Although SMADs 2, 3, and 4 have been proposed to work together to drive transcription, it is clear from these reports that each SMAD protein has a unique role in Th17 differentiation.

SMAD7 expression is increased in lesional tissue isolated from patients with UC and CD, whereas phosphorylation of SMADs 2 and 3 is reduced as compared to non-IBD controls (128), suggesting a dominant role for SMAD7 in IBD. Blockade of SMAD7 activity with an anti-sense oligonucleotide enhanced SMAD2 activation and reduced inflammatory cytokine production in intestinal explants (128) and severity of colitis in mice sensitized and treated with TNBS or oxazolone (29). Furthermore, SMAD7-overexpressing T cells enhanced development of colitis in a RAG adoptive transfer model of colitis even in the presence of co-transferred T regulatory cells (129). Interestingly, SMAD7 transgenic CD4^+^ T cells isolated from the intestines of these mice are not enriched for IL-17-producing cells, but instead have more IFN-γ-producing Th1 cells and have a lesser capacity to express FOXP3. These data are consistent with previous reports that demonstrated that TGF-β signaling in T regulatory cells induced FOXP3 which in turn limits SMAD7 expression (124). As a whole, these data indicate that an imbalance in T cell-intrinsic SMAD signaling does not promote Th17 differentiation *in vivo*, but rather enhances the ability of T cells to avoid Treg-mediated suppression.

#### RORγt

An immune-specific isoform of retinoic acid receptor-related orphan nuclear receptor gamma (RORγt), a type of nuclear hormone receptor (NHR), is critical for the differentiation of Th17 cells (95). RORγt is induced by both IL-6/STAT3 and in some cases TGF-β, two cytokines that drive Th17 differentiation (130). Currently, there has been no identified IBD-associated *RORC* SNPs suggesting that while it is critical for Th17 differentiation, variations in this gene are not associated with enhanced disease.

Nuclear hormone receptors, like RORγt, are prime candidates for therapeutic intervention based on their mechanism of action where binding of a particular ligand controls their transcriptional activity. Natural ligands for RORγt have been recently identified as being a cholesterol biosynthetic intermediates (CBIs) and deletion of enzymes that generate these CBIs appear to be involved in Th17 differentiation (131). There has been a great deal of interest in generation of small molecule inhibitors that would be able to block or displace binding of natural RORγt ligands to therapeutically inhibit Th17 responses during IBD. Withers et al. (132) demonstrated that therapeutic delivery of an oral-available Rorγt inhibitor (GSK805) was able to significantly reduce disease in a number of murine IBD models. Interestingly, GSK805 treatment was more effective in treating disease that IL-17 blockade or deficiency and was not marked by compensatory increases in IFN-γ production as observed in studies mentioned above. These data suggest that either RORγt small molecule inhibitors target additional RORγt-expressing cells types that contribute to inflammation (i.e., ILC3s) or inhibit other Th17-associated functions besides IL-17 production.

## Th22 Cells

Th22 cells are characterized by their production of IL-22, but not IFN-γ or IL-17. Differentiation of Th22 cells occurs in the presence of IL-6, TNF-α, and IL-1β, and suppressed by TGF-β (Figure 1) (133, 134). Similar to Th17 cells, IL-21 also can enhance the differentiation of IL-22-producing T cells (135). Although Th22 cells do not produce IFN-γ, they express high levels of T-bet that is critical for their differentiation (136). Furthermore, while expression of the aryl hydrocarbon receptor (AhR) TFs is low in Th22 cells as compared to Th17 cells, it is also critical for IL-22 production from CD4 T cells (133, 136).

### Th22-Associated Cytokines

#### Interleukin-22

Interleukin-22 is a member of the IL-10 family of cytokines and binds to IL-22R which is complex of IL-22R1 and IL-10R2 that signals primarily through STAT3 (137). IL-22 is elevated in both mice and humans with IBD (138, 139) and is known to have a protective effect against gut inflammation, tissue damage, and bacterial infection (136, 140–142). IL-22 producing Th cells were significantly reduced in inflamed tissues from UC patients and were replaced by IL-17-producing Th17 cells, suggesting that the balance of inflammatory and anti-inflammatory cells is disrupted during IBD.

In IBD, IL-22 ameliorated intestinal inflammation in a STAT3-dependent manner that correlated with enhanced mucus production by colonic epithelial cells (143). Mice deficient in IL-22 are susceptible to *C. rodentium* infection (136) and mice that lack STAT3 specifically in T cells exhibited enhanced susceptibility to infectious colitis during *C. rodentium* infection that was rescued by intestinal overexpression of IL-22 (144). Furthermore, adoptive transfer of Th cells cultured under Th22 conditions was sufficient to protect from *C. rodentium* infectious colitis (136). In both a DSS and RAG adoptive transfer model of colitis, IL-22-deficient mice or mice receiving IL-22-deficient Th cells exhibited enhanced weight loss and colon inflammation as compared to controls indicating that IL-22 has a protective role in multiple models of murine colitis (145, 146). Together, these data suggest that Th22 cells are a relevant protective source of IL-22 during intestinal inflammation.

Interleukin-22 mediates its protective properties by inducing production of mucus and AMPs that have direct anti-microbial activity (141, 147). In addition, injection of IL-22 into mice induced the expression of LPS binding protein by hepatic cells thereby increasing the capacity to neutralize systemic LPS and reduce inflammation (138).

#### IL-22 Binding Protein (IL-22BP)

IL-22 binding protein exists as soluble inhibitory receptor and it is expressed at high levels in the PP and colon by DCs in the steady state and by CD4^+^ T cells during inflammation (146, 148–150). IL-22BP has higher affinity than IL-22R1 and competes for IL-22 binding with the cell-associated receptor, and interferes with the protective role of IL-22. Both UC and CD patients exhibit increased IL-22BP expression and IL-22BP^+^ cells in inflamed intestinal tissue as compared to healthy controls (149). Importantly, transfer of IL-22BP-deficient Th cells into either IL-22BP-sufficient or -deficient RAG mice resulted in less disease as compared to mice receiving WT Th cells, indicating that Th cell-derived IL-22BP was the biologically dominant source for pathogenic IL-22BP production during IBD (149). In future studies, it will be important to determine what Th cells are producing IL-22BP (i.e., Th1 and Th17) or if these cells are a unique Th subset. Furthermore, the factors that result in IL-22BP production by Th cells is unknown. Understanding of this pathway will have clear implications for treatment of IBD.

### Th22-Associated TFs

#### Aryl Hydrocarbon Receptor

STAT3 signaling and T-bet are required for the differentiation of Th22 cells (136) and the role of these factors in IBD have been discussed above in terms of Th17 and Th1 differentiation, respectively. Although Th22 cells do not express elevated levels of AhR as compared to Th17 cells, it is critical for their differentiation (136). As Th22 cells are associated with protection against intestinal disease, it is not surprising that AhR is also associated with protection from IBD. Patients with IBD exhibit significantly higher IL-22 and AhR expression as compared to healthy individuals (151), presumably to counteract enhanced inflammation within the tissue. In mice, transfer of AhR-deficient Th cells in a RAG model of colitis induced more severe disease as compared to mice that received WT Th cells (135). In a murine TNBS colitis model, an AhR antagonist induced more severe colitis by suppressing synthesis of IL-22. By contrast, treatment of AhR agonist 6-formylindolo(3, 2-b)carbazole (FICZ), a tryptophan derivative, reversed relapsing TNBS and DSS-induced colitis (151). Mechanistically, tryptophan metabolites can alter the intestinal microbiota in favor of microbes that induce AhR production and AhR-dependent IL-22 transcription (152). As a whole, these data indicate that AhR expression in Th cells is required for IL-22 production and protection from IBD.

## Th2 Cells

Th2 cells classically function to provide anti-parasite immunity, but are also known to be effector cells in asthma and differentiate in response to IL-4 (153). IL-4 signaling in Th2 cells leads to activation of the receptor-associated signaling molecule STAT6 and downstream induction of the Th2 defining TF GATA3 (Figure 1) (153). GATA3 is able to further polarize the differentiation of Th2 cells through a positive autoactivation pathway (154) and can also convert committed Th1 cells can to Th2 cells when ectopically expressed (155). While Th1 cells are indicative of CD, Th2 or Th2-like cells are more associated with UC. This Th1/Th2 paradigm, although controversial, has been recently supported by the development of an equation that can predict CD vs. UC based on cell populations with 83% accuracy (156).

### Th2-Associated Cytokines

#### Interleukin-4

The role of IL-4 in perpetuating IBD is controversial. T cells isolated from UC biopsies do not exhibit significant production of IL-4, which has shown to be vital in the differentiation of Th2 cells and their defining cytokine (157). Similarly, *IL4* mRNA expression in intestinal mucosa in both CD and UC patients was undetectable (158). IL-4, in combination with IL-10 (another cytokine enriched in Th2 cells), has also been shown to synergistically inhibit the pro-inflammatory cytokines TNF-alpha and IL-1β that are associated with IBD (159). Furthermore, SNPs present in the IL-4 gene are overrepresented in UC patients and are presumably loss of function mutations, suggesting a possible regulatory role of these cytokines in IBD patients (Figure 1; Table 2) (69). However, anti-IL-4 treatment of mice undergoing oxazolone-induced colitis or TCRα^−/−^ adoptive transfer model prevented the majority of disease (31, 43, 160). These data indicate that IL-4 may have anti- or pro-inflammatory roles based on the particular type of IBD or mouse model used.

#### Interleukin-5

Stimulated lamina propria (LP) T cells isolated from colonic biopsies from UC patients had increased expression of IL-5 compared to CD and control patients (161). An assessment of cytokine transcripts in UC and CD patients found IL-5, IL-13, IL-15, and IL-33 mRNA levels to be increased in UC patients (162). Although IL-5 is produced in these tissues, its exact contribution to UC is still unclear. IL-5 is a potent inducer of eosinophils from the bone marrow and there is some evidence that elevated IL-5 levels in UC promotes the recruitment of eosinophils into the inflamed intestine (163, 164). The role of these eosinophils in UC, however, remains unclear.

#### Interleukin-13

Similarly, LP mononuclear cells from UC produced much larger amounts of IL-13 than CD or control patients (33). However, the source of IL-13 was not always T cell derived. In human studies, both LP Th cells and invariant natural killer T cells accounted for the majority of the IL-13 production (161, 165). IL-13 is also produced in mice by intestinal ILCs (166, 167). Interestingly, IL-13 signaling through the once thought “decoy” IL-13Rα2 receptor induces TGF-β and fibrosis of intestinal tissue in a chronic model of TNBS colitis (26, 27). Given the apparent importance of IL-13 in both human IBD and murine model studies, IL-13-specific antibodies (anrukinzumab and tralokinumab) have been trialed as therapy for UC patients. Unfortunately, anrukinzumab did not show significant therapeutic effect in UC patients with active disease (168). Tralokinumab showed some improved clinical remission rates and mucosal healing, but did not have significant clinical response when compared to placebo, which may indicate limited therapeutic benefit (169).

### Th2-Associated TFs

#### Signal Transducer and Activator of Transcription 6

STAT6 is activated by the cytokines IL-4 and IL-13 and binds a number of IL-4-responsive promoters (170). Activated STAT6, as measured by phosphorylation, was also enhanced in intestinal tissues take from UC patients and correlated with disease severity (171). There is also one reported SNP in the STAT6 gene that is associated with CD (Figure 1; Table 2) (68). In models of UC (e.g., DSS and oxazolone-induced UC), STAT6 can play an important role in IBD pathogenesis through changes in inducible NO synthase or tight junction proteins and Th2 cytokine production (20, 172). However, STAT6 was found to be dispensable in a TCRα^−/−^ colitis model (173).

#### GATA3

STAT6 binds the promoter and activates transcription of GATA3; GATA3 can subsequently induce its own expression and inhibit Th1 production. Thus, GATA3 is a lineage-defining factor for Th2 cells. GATA3 was expressed at higher levels in colonic tissue from UC patients compared to ileal CD patients and correlated with disease severity. Furthermore, UC patients had an increased number of mucosal CD4^+^/GATA^+^ cells (35). This has also been shown in pediatric UC patients, those with active disease had increased expression of mucosal *GATA3* compared to age-matched controls (174). In a murine models of UC (DSS and oxazolone-induced UC), overexpression of GATA3 accelerates acute colitis in contrast to overexpression of T-bet or RORγt (21, 35). Furthermore, T cell-specific GATA3-deficient mice were resistant to oxazolone-induced disease and treatment with hgd40 DNAzyme that cleaves *GATA3* mRNA also exhibit a significant decrease in disease severity (35). Currently, SB012, which contains hgd40, is being investigated for use in UC patients (https://www.clinicaltrials.gov/show/NCT02129439).

#### Others

c-MAF is a TF that is associated with Th2 and Th17 differentiation and aids in both IL-4 and IL-17 production (175, 176). CD and UC patients have increased numbers of c-MAF^+^ T cells in the inflamed intestine. Interestingly, c-MAF^+^ cells were also T-bet^+^, a Th1-associated factor, suggesting that these cells may have a Th1-like phenotype (177). Furthermore, c-MAF overexpressing naïve CD4 T cells were unable to induce colitis as compared to WT controls. However, c-MAF overexpression within memory/effector CD4 T cells, normally containing T regulatory cells, augmented colitis when co-transferred with naïve Th cells (177). Deletion of c-MAF in T regulatory cells also limited their ability to produce the anti-inflammatory cytokine IL-10 and regulate microbiota-specific Th cells (178). These data indicate that there is a fine balance in c-MAF expression in T regulatory cells that controls their regulatory capacity.

The TF NFAT is also involved in Th2 differentiation and NFAT family member expression was enriched in the inflamed intestine during IBD (179). A gene controlling NFAT nuclear translocation was identified as a key IBD susceptibility gene in human IBD GWAS studies (180). Furthermore, genetic deletion of NFATc2 results in reduced disease in an oxazolone model of murine colitis by modulating the ability of Th cells to produce IL-6, a cytokine normally associated with Th2 cells (181).

## Th9 Cells

Interleukin-9 producing T cells were initially thought to be a subset of the Th2 population. This changed after the finding that TGF-β, together with IL-4 reprogram Th2 cells to become Th9 cells that secrete IL-9 and IL-10 and have a unique transcriptional profile (Figure 1) (182, 183). In these cells, IL-9 production is under the control of PU.1, STAT6, BATF, GATA3, and IRF4 (183, 184). TGF-β, in particular, stimulates the production of TF PU.1, which in turn induces IL-9 expression (185).

### Th9-Associated Cytokines

#### Interleukin-9

Recent studies both of CD and UC patients have shown IL-9 production to be enhanced in these disease states and correlates with endoscopic Mayo scores (34). Further studies have also revealed that in UC patients, activated peripheral blood lymphocytes produced increased amounts of IL-9 (186). Clinically, patients with increased serum IL-9 levels have been shown to have a worsened prognosis (187) and higher levels of systemic IL-9 were also associated with cachexia and lower hemoglobin concentrations in IBD patients (188).

In mouse studies, mice with oxazolone-induced colitis exhibited increased IL-9 expression and IL-9-deficient mice were resistant to development of colitis (34). Interestingly, therapeutically targeting IL-9 with a neutralizing antibody in these studies also significantly reduced disease over controls, and this was also verified in a DSS model of UC (22). In a TNBS-induced colitis model, IL-9-deficient mice were also resistant to colitis development and suggested that IL-9 may have a role in regulating intestinal barrier function (28). In additional studies using the RAG adoptive transfer model of colitis found that when IL-9-producing T cells were transferred into RAG 1-deficient mice, the mice exhibited increased degree of colitis (189). As compared to other cytokines that have model-dependent protective or pathogenic roles in IBD (i.e., IFN-γ and IL-17), IL-9 appears to have a consistent pathogenic role across disease models making it a potential druggable target in IBD.

Tofacitinib, one of the newest approved therapies for IBD, is a small molecule Janus kinase (JAK) inhibitor that has shown promising therapeutic potential in UC (190). The JAK family includes intracellular tyrosine kinases that activate STATs to control multiple cytokines including IL-9. However, IL-9 is one of the many cytokines that are inhibited; perhaps newer therapies with more targeted IL-9 activity may be beneficial in patients who have failed other forms of treatment.

#### Interleukin-10

Th9 cells also produce IL-10, which is classically known to be immunosuppressive (184). However, as above, experiments by Dardalhon et al. showed that transferring IL-9 and IL-10 double positive cells into RAG 1-deficient mice worsened colitis in these mice (189). However, IL-10 and its role in IBD has been studied extensively. IL-10-deficient mice irrefutably develop colitis spontaneously and pathologically resemble human IBD (191). The role of IL-10 in relation to Th9 cells, however, needs to be further elucidated.

### Th9-Associated TFs

#### PU.1

PU.1 is required for Th9 development and is produced more in these cells compared to the other subsets (185). In Th9 cells, PU.1 binds to the *Il9* promoter and alters histone acetylation (192). In human IBD, the number of PU.1^+^ Th cells is higher in mucosal biopsies of patients with active IBD (34). This was also shown using immunohistochemistry to assess the density of PU.1 expressing cells in both human UC and murine DSS colitis (22). Moreover, mice with PU.1-deficient T cells had diminished pathology in the oxazolone model (34).

#### Others

STAT6, GATA3, and SMAD proteins are also required for Th9 differentiation and may be involved in Th9-mediated colitis. Indeed, IL-9 is significantly reduced in oxazolone-induced colitis when GATA3 is deficient in T cells (35). However, the role of STAT6 and SMADs on IL-9 production in colitis has not been examined. IRF4 is also required for differentiation of Th2, Th9, and Th17 cells (193–195) and is required for inflammation in a RAG adoptive transfer model of colitis (196, 197). Therefore, a number of TFs that play a role on other Th lineages can also impact Th9 function during intestinal inflammation.

## Outlook on IBD Therapy

Over the last several decades, researchers have generated a tremendous amount of information regarding how the CD4^+^ Th response influences the outcome of IBD in both mice and humans. This has already led to the development of a number of biologics (i.e., anti-TNF) that are effective in causing at least temporary remission from disease. However, these therapies are only effective in causing endoscopic and microscopic remission in a subset of patients. In this review, we have highlighted a number of instances where depletion or deletion of particular Th-associated cytokines can have deleterious effects. In the majority of these instances, there are compensatory immune mechanisms that either render treatment ineffective or make disease considerably worse. Clearly, more work must be done to identify these compensatory mechanisms and be able to predict them in both murine models of disease and in human patients. Broad-scale successful treatments of IBD will likely require targeting multiple arms of the Th cell response to account for compensating mechanisms that may be unique to each individual. These personalized treatments might include bi-specific antibodies or multiple biologics used in conjunction to account for predicted compensatory responses.

There are, however, some inherent drawbacks to this “multi-faceted” approach. Targeting multiple immune mechanisms will likely occur at increased cost to the patient. CD treatment is already ~3× more expensive than UC therapy with ~64% of that cost being driven by anti-TNF therapy (198), a cost that will likely increase when multiple biologics are used. One approach to this problem is to utilize a broader pan-Th inhibitor that might target multiple cytokines that are produced by a number of Th subsets. As the majority of these cytokines signal through cytokine receptor-associated JAKs, JAK inhibitors are an attractive target for limiting autoimmune inflammation. The JAK inhibitor tofacitinib has been approved for treatment of rheumatoid arthritis and has shown promise in phase 3 clinical trials in IBD (199). Beyond cost, targeting multiple arms of the immune system may also lead to increased susceptibility to infection or cancer. Although a number of Th-associated cytokines are involved in IBD pathogenesis, almost all of these have evolved for protection from infection and for regulating gut homeostasis and microbiota. Patients on anti-TNF therapy are more susceptible to a number of infections and development of particular types of tumors (200, 201). It is easy to imagine that combination of anti-TNF therapy with anti-IL-23 or a JAK inhibitor would further increase susceptibility and thereby limit the efficacy or desirability of treatment. Ideally, future treatments would target offender-only molecules that do not also play protective or homeostatic roles in the immune system. A recent study by van Unen et al. (202) used high parameter time of flight mass cytometry (CyTOF) to identify a number of novel immune cell populations that correlate with disease in IBD patients. This type of data will be instrumental in identifying possible “offender-only” immune cell populations that may be targeted therapeutically without hampering normal host immune responses.

## Author Contributions

TI, SP, MK, and MO wrote the manuscript and designed figures and tables.

## Conflict of Interest Statement

The authors declare that the research was conducted in the absence of any commercial or financial relationships that could be construed as a potential conflict of interest. The handling Editor declared a past co-authorship with one of the authors [MK].
